# The PU‐PROM: A patient‐reported outcome measure for peptic ulcer disease

**DOI:** 10.1111/hex.12575

**Published:** 2017-06-21

**Authors:** Na Liu, Jing Lv, Jinchun Liu, Yanbo Zhang

**Affiliations:** ^1^ Department of Health Statistics School of Public Health Shanxi Medical University Shanxi Medical University molecular imaging precision medicine Collaborative Innovation Center Taiyuan Shanxi Province China; ^2^ Department of Gastroenterology The First Hospital Shanxi Medical University Taiyuan Shanxi Province China

**Keywords:** classical test theory, differential item functioning, item response theory, patient‐reported outcome, peptic ulcer, reliability, validity

## Abstract

**Objective:**

Patient‐reported outcome measure (PROM) conceived to enable description of treatment‐related effects, from the patient perspective, bring the potential to improve in clinical research, and to provide patients with accurate information. Therefore, the aim of this study was to develop a patient‐centred peptic ulcer patient‐reported outcome measure (PU‐PROM) and evaluate its reliability, validity, differential item functioning (DIF) and feasibility.

**Method:**

To develop a conceptual framework and item pool for the PU‐PROM, we performed a literature review and consulted other measures created in China and other countries. Beyond that, we interviewed 10 patients with peptic ulcers, and consulted six key experts to ensure that all germane parameters were included. In the first item selection phase, classical test theory and item response theory were used to select and adjust items to shape the preliminary measure completed by 130 patients and 50 controls. In the next phase, the measure was evaluated used the same methods with 492 patients and 124 controls. Finally, we used the same population in the second item reselection to assess the reliability, validity, DIF and feasibility of the final measure.

**Results:**

The final peptic ulcer PRO measure comprised four domains (physiology, psychology, society and treatment), with 11 subdomains, and 54 items. The Cronbach's α coefficient of each subdomain for the measure was >0.800. Confirmatory factory analysis indicated that the construct validity fulfilled expectations. Model fit indices, such as RMR, RMSEA, NFI, NNFI, CFI and IFI, showed acceptable fit. The measure showed a good response rate.

**Conclusions:**

The peptic ulcer PRO measure had good reliability, validity, DIF and feasibility, and can be used as a clinical research evaluation instrument with patients with peptic ulcers to assess their condition focus on treatment. This measure may also be applied in other health areas, especially in clinical trials of new drugs, and may be helpful in clinical decision making.

## 1 INTRODUCTION

Peptic ulcer is defined as an ulcer occurring in a region that touches gastric acid and pepsin, and usually refers to a gastric or duodenal ulcer. Although peptic ulcers have a very low mortality rate, they can have complications such as haemorrhage and perforation if not treated in time. They can cause significant physical pain to patients and increase financial and service burdens. One report found that the lifetime prevalence of peptic ulcer disease in the general population has been estimated to be about 5%‐10%, and incidence 0.1%‐0.3% per year.^1^, ^2^, ^3^ Although peptic ulcers are a benign, non‐fatal disease, they do result in lost productivity and associated economic loss.

Distress caused by peptic ulcers includes psychological, social and behavioural problems, which may interfere with a patient's ability to fully participate in their health care, and manage their illness and its consequences. The widely accepted bio‐psycho‐social medical model includes quality of life (QoL), which is the general well‐being of individuals and societies, outlining negative and positive features of life. It is understood to be a multidomain concept with physical, psychological and social components, however which is insufficient to assess the patients treatment‐related effects. Besides, some of the symptoms associated with treatment, such as nausea and vomiting, are frequently underreported by clinicians, even when data are prospectively collected within clinical trials. Only relying on the results of clinical examination and the judgement of experts without patients’ perception could indirectly lead to misdiagnosis and medical accidents because of the different opinions between doctors and patients. Developing a patient‐centred measure tool can enhance the self‐efficacy of patients to recognize and report symptoms of deteriorating conditions, improve patient ‘s self‐efficacy, identify and report the deterioration of symptoms and able to actively contribute to the management of chronic diseases.^4^, ^5^ Patient‐reported outcomes (PROs) have been demonstrated to be a valid, reliable, feasible and precise approach to evaluate clinical research and enables symptoms that are missed by clinicians to be detected.

The International Society for Pharmacoeconomics and Outcomes Research (ISPOR), the European Regulatory Issues on Quality of Life Assessment Group (ERIQA), the United States Food and Drug Administration (FDA)^6^ and the International Society for Quality of Life Studies (ISQOL) recommend that clinical efficacy evaluation should include outcomes provided by patients and proxy respondents. Patient‐reported outcomes (PROs) are a central aspect.^7^ A PRO is a measurement of any aspect of a patient's health status that originates directly from the patient (i.e, without the interpretation of the patient's responses by a physician or other person).^8^ A PRO can directly reflect the influence of disease in a patient, help in the treatment of that patient and help to establish good communication between the patient and medical staff in determining treatment efficiency. In addition, a PRO can help to explain clinical outcomes and treatment decisions made.

In recent years, many effective and reliable measures for the digestive system have been developed internationally and applied in practice. Commonly used measures include the Sickness Impact Profile (SIP),^9^ the Nottingham Health Profile (NHP)^10^ and the Quality of Well‐being (QWB).^11^ The Medical Outcomes Study Short Form‐36 (SF‐36) is the most well‐known and widely accepted measure.^12^ Specific health‐related QoL (HRQoL) measures commonly used include the Quality of life in peptic diseases (QPD) questionnaire,^13^ Peptic ulcer diseases questionnaire (PUDQ), Ulcer esophagitis subject symptom (UESS), Quality of life in duodenal ulcer patients (QLDUP).^14^

Many studies have been performed on the demographic and clinical characters and health‐related quality of life in the disease‐specific patients based on all those measures. However, these scales all have a specific focus, and cannot fully reflect the actual situation of patients with peptic ulcers. Therefore, in this study, we aimed to develop a PRO measure for peptic ulcer patients (PU‐PROM) that (I) was developed from the perspective of patients and could be widely applied in evaluations of clinical curative effect; (II) is established across four domains (physical, psychological, social and treatment) to comprehensively reflect health status and quality of life in patients with peptic ulcers; (III) can be specifically used to report the clinical outcomes of patients with peptic ulcers; (IV) comprised items that were easy to understand and answer (responses on 5‐point Likert scale).

## 2 METHODS

### 2.1 Ethics statement

The study protocol and the PU‐PROM were reviewed and approved by the Medical Ethics Committee of Shanxi Medical University.

### 2.2 Study population and design

Based on the principles of the United States FDA, we performed a comprehensive review of the literature and related measures, conducted semi‐structured interviews with patients with peptic ulcers and consulted relevant experts. We extracted and integrated information from these sources to form a theoretical framework and an initial item pool. The interview participants were 10 patients with peptic ulcers with a consistent disease distribution by sex and age, including six men and four women aged 40.01±9.81 years. The six experts consulted included three chief peptic ulcer physicians, one psychologist, one sociologist and one ethics expert. Based on the patient interviews and expert consultation, we debugged and modified the expressions of items in the initial item pool.

For the first item screening stage, 200 participants were sampled from eight hospitals at different levels in Shanxi Province, China. This included 150 patients with peptic ulcers and 50 controls. Completed questionnaires were examined using classical test theory (CTT) and item response theory (IRT), to select and adjust the item pool, and a preliminary measure was developed. This was followed by a formal investigation, with 550 patients with peptic ulcers and 150 controls, using the same method to reduce the items and form the final measure. Finally, the reliability, validity, differential item functioning (DIF) and feasibility of the measure were verified.

### 2.3 Development of the PU‐PROM

**Figure 1:**
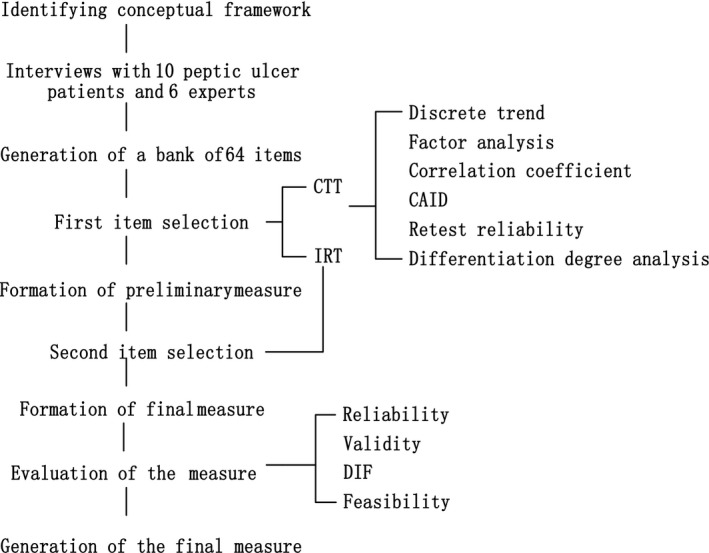
Developmental process flow chart

The PU‐PROM was developed in three phases: (i) conceptual framework construction and initial item generation; (ii) formation of the final measure by two item selection process based on the CTT and IRT; and (iii) validation of the PU‐PROM. Phase 1 involved a qualitative analysis, whereas the other two phases used quantitative analyses. A flow chart of this developmental process is shown in Figure 1.

#### 2.3.1 Step 1: Item generation

##### Identifying the conceptual framework and initial item content

We searched for related literature of PRO measures to build the conceptual framework of PU‐PROM. The theoretical framework was established including four domains and 12 subdomains: physiology (subdomains: physical symptoms, independence and physical status); psychology (subdomains: work stress, anxiety, depression and fear); society (subdomains: social support and social adaptation); and treatment (subdomains: compliance, degree of satisfaction and availability). Then, the patients were interviewed to understand the main symptoms, the influence of the psychological and social, and the evaluation of treatment satisfaction; all of the information were collected and collated to form the item pool. Next, the item pool was revised based on discussions with the six experts and 10 patients. They put forward items that ambiguous in words and difficult to understand should be deleted, and some items should be added. Subsequently, an initial version of the measure was developed, using four domains, 11 subdomains (independence was excluded from the physiology domain) and 64 items.

#### 2.3.2 Step 2: Item selection

##### Sampling survey

We selected a sample comprising 150 peptic ulcer patients and 50 controls from the eight participating hospitals in Shanxi Province. Patients who were diagnosed with a definite peptic ulcer, who were fully competent and who volunteered to participate were included in this study. Patients were excluded if they had deficiencies in language or cognitive abilities that meant they could not understand or complete the questionnaire; mental illness; or disturbance of consciousness. The controls did not have peptic ulcers, malignant tumours or mental illness, and had a similar age distribution to the patient group.

In the process of evaluating the patients’ completed questionnaires, 20 were invalid and 130 were valid, giving a valid response rate of 87%. The valid response rate for the controls was 100%.

Reponses to all items were on a 5‐point Likert scale, where recorded as 0‐4 points. The 5‐point scale form was most commonly chosen as the easiest to complete, and item omission was least frequent with this form. Nagata suggests that the 5‐point scale is most useful for measuring health status.^15^ The measure contained positive items and negative items. Positive items were scored as the original score plus one and negative items were scored as five minus the original score. Missing data were tested by Little's Missing Completely at Random test, and the *P*‐value was <.001.^16^ Items considered as missing at random were imputed based on the expectation‐maximization algorithm.^16^

##### Statistical methods for item selection

The item reduction for the preliminary measure was based on CTT and IRT. CTT includes discrete trend, factor analysis, correlation coefficients, Cronbach's α if an item is deleted, retest reliability and differentiation degree analysis. These methods were combined with specialized knowledge to assess the items. Items selected at by least five methods were kept, although this meant that other methods might have suggested that the item be removed. The final version of the preliminary measure comprised four domains, 11 subdomains and 54 items (10 items were deleted).

##### CTT

###### Discrete trend

The scores of all items (derived from the 5‐point Likert scale) could be regarded as similar to normal processing. A low discrete trend indicates people inclined to select the same answer, and the item has poor evaluation ability. We used the standard deviation (SD) to measure the discrete degree of items. Items with a low SD (<1.0) were deleted.

###### Factor analysis

We used principal component factor analysis, with the largest orthogonal rotation according to the eigenvalue and the variance contribution rate of an item. The value for the Kaiser‐Meyer‐Olkin measure of sampling adequacy was >0.5. Items with low factor loading (<0.4) and cross‐measurement with more than one other item were deleted.

###### Correlation coefficient

Items were filtered by representativeness and independence, which showed the item satisfied the purpose of the PROM. An item that showed a low correlation coefficient (<0.6) in relation to its subdomain was deleted.

###### Cronbach's alpha if item was deleted (CAID)

The method used internal consistency to choose items and ensure homogeneity.^17^ The internal consistency of items was evaluated by calculating the CITC and CAID values (Cronbach's α), when the CITC value was more than 0.45, which showed the item has highly contribution to the measured construct. The CAID values determined which item has highly contribution to the reliability of the PROM. Whether the Cronbach's α coefficient showing a big increment after an item was removed indicated that item was influential for internal consistency and it should be deleted.

###### Retest reliability

Retest reliability reflects stability and consistency across time. We selected 4 days as the retest interval and calculated the correlation coefficient of the item score across two surveys. Items with low correlation coefficients (<0.6) were deleted.

###### Differentiation degree analysis

Whether the item could not make a distinction between different objects that should be deleted. We compared each item score for the patient and control groups by performing independent two‐sample *t*‐tests (α=0.05). Items with no statistical difference were deleted.

##### IRT

Item response theory is a nonlinear model used to select items and test construction. It establishes a functional relationship between a participants’ reaction to the item and their ability. This relationship is reflected by an item characteristic curve (ICC). Items were assessed using Multilog 7.03 with a grade response model. Each item's parameters of discrimination (α) and difficulty (b) were estimated. In general, items with a discrimination value of <0.4 should be deleted. Difficulty was divided into four grades (b1, b2, b3 and b4) ranging from −3 to 3. Items outside this range should be considered for deletion.

##### Second sampling survey

To verify the measure, we recruited 550 patients and 150 controls from the same eight hospitals, but only 492 and 124, respectively, were available to participate in the study. The response rate met the requirements, and the number of participants satisfied Nunnally's proposal.^18^ We used the same methods as used for the preliminary measure to revise the items.

Classical test theory and item response theory were used for item reselection. Combined with professional knowledge, we used at least five methods to evaluate the items. Three item evaluation methods were discarded. The results indicated that all 54 items should be kept to form the final measure.

#### 2.3.3 Step 3: Validation of the measure

Finally, we using the data obtained from these 492 patients as well as 124 control participants to examine the reliability, validity, DIF and feasibility of the final measure.

##### Reliability

Reliability refers to the consistency of the test results; the higher the measured value of homogeneity, the better the reliability.^19^ Cronbach's α coefficient and retest reliability are frequently used in reliability analyses. Cronbach's α coefficient is the most commonly used method. In this study, we calculated every subdomain's Cronbach's α coefficient. And generally, the α value should be more than 0.7. Retest reliability reflects the stability and homogeneity across time. And it is generally believed that the correlation coefficient should be more than 0.6.

##### Validity

Validity analysis evaluates the validity of a questionnaire. This involves content validity, construction validity and discriminant validity. Content validity reflects the degree to which the selected items represent the expected content.^19^ Construction validity (or structure validity) examines whether the multi‐index measurement is a professionally ideal structure, testing the structure from clinical and common sense perspectives. A measure with good construction validity can obtain true latent trait of subjects during measurement. We used confirmatory factor analysis (CFA) to build a measurement model between the item and the subdomain that included the item. We used LISREL 8.70 software (Scientific Software International. Inc. 7383 North Lincoln Avenue, Suite 100 Lincolnwood, IL 60712‐1704) for the CFA.^20^ In addition, the evaluation of the fit effect of the model for every domain was also used multiple indexes, commonly fit index including: GFI, RMR, NFI, NNFI, IFI, CFI.^21^ Discriminant validity reflects small changes across different populations and different times. It can reflect the different trait of selected subjects. Discriminant validity was assessed by comparing the mean score of the patients and the controls to ensure whether each subdomain correctly distinguish the two types of people. Generally, we used a simple independent two‐sample *t*‐test to compare patients and controls. When the *P*‐value was <.05, we considered the difference to be statistically significant, and the measure to have a good degree of differentiation.

##### DIF

As a test result has personal, social and political ramifications, it should be reliable, valid and fair.^22^ To investigate whether a test item is fair among members of different subgroups such as males and females and majority groups and minority groups, a plethora of research on DIF has been conducted.^23^, ^24^ This verified the quality of the questionnaire and ensured the validity and fairness of the measure. DIF is generally divided into two types of uniform DIF and non‐uniform DIF. Uniform DIF is present when an item differs across groups in item difficulty parameters, while non‐uniform DIF is present when an item differs across members of different subgroups in item discrimination parameters.^25^ The MACS model identifies non‐uniform DIF and uniform DIF using unidimensional multistage scoring. If there is no DIF in the items of all subdomains, we further confirmed it by the comparison of nested models. Comparing the chi‐square difference between the “measurement equivalence model” and the “baseline model,” if the difference was not significant, the items of the subdomains did not exist DIF; if there is DIF in the items of all subdomains, we need to compare the chi‐square difference between the “measurement equivalence model” and the “partial measurement equivalence model”; if the difference has statistical significance, indicating that the items exist DIF.

##### Feasibility

Feasibility is used to reflect the degree of acceptability of a measure. This is characterized by acceptance rate, response rate and completion times. In general, the recovery rate of the questionnaires should more than 85%, and the response rate also should more than 85%. In addition, time for each person answered should be controlled within 15 minutes.

### 2.4 Statistical software

Data analyses were performed with spss, version 16.0 (SPSS for Windows, Version 16.0. Chicago, SPSS Inc.), Multilog 7.03 and LISREL 8.70.

## 3 RESULTS

### 3.1 Participant characteristics

In the first survey with the preliminary measure, 130 patients with peptic ulcers aged 41.25±5.97 years and 50 controls aged 40.00±5.49 years completed the measure. In the formal survey, 492 patients aged 41.84±8.81 years and 124 controls aged 41.35±8.35 years completed the measure. Tables 1 and 2 present the participants’ characteristics, and in the first item pool reduction phase, and the revaluation and validation phase, respectively.

**Table 1:** Baseline data for participants in sample survey

Variables	Peptic ulcer	Control	*t*/χ^2^	*P*
Age (years)	41.25±5.97	40.00±5.49	1.290	.199
Gender				
Male	65	31	2.089	.148
Female	65	19		
Height (cm)	165.90±4.97	165.52±4.70	0.466	.642
Weight (kg)	64.08±7.84	63.64±8.03	0.478	.634
Drinking				
Never	37	16	0.616	.959
Quit	62	23		
Occasional	29	10		
Always	2	1		
Smoking				
Never	14	8	2.121	.548
Quit	47	19		
10 branches/d	44	12		
10 branches/d~	25	11		

**Table 2:** Baseline data for participants in formal survey

Variables	Peptic Ulcer	Control	*t*/χ^2^	*P*
Age (years)	41.84±8.81	41.35±8.35	0.555	.579
Gender				
Male	283	82	3.040	.081
Female	209	42		
Height (cm)	164.87±2.26	165.53±5.82	−1.230	.219
Weight (kg)	64.96±7.29	66.37±6.96	−1.948	.052
Drinking				
Never	142	31	1.041	.791
Quit	228	58		
Occasional	110	32		
Always	12	3		
Smoking				
Never	112	33	1.577	.665
Quit	228	50		
<10 branches/d	116	31		
10 branches/d~	36	10		

### 3.2 Item generation

Through a large number of literature, expert consultation and patient interviews, we conducted a conceptual framework consisted of four domains, 12 subdomains and a pool of 77 items. Next, we selected items based on interviewing 10 patients with peptic ulcer and consulting six relevant experts to ensure that all the remaining items are easy to understand and related to the topic. So, one domain and 18 items describing atypical symptoms and overlap with each other were deleted, and five items were added. Therefore, there are four domains, 11 subdomains and 64 items were generated for the initial peptic ulcer PRO measure. The items are described in Additional file 1: Appendix 1.

### 3.3 Item selection

**Figure 2:**
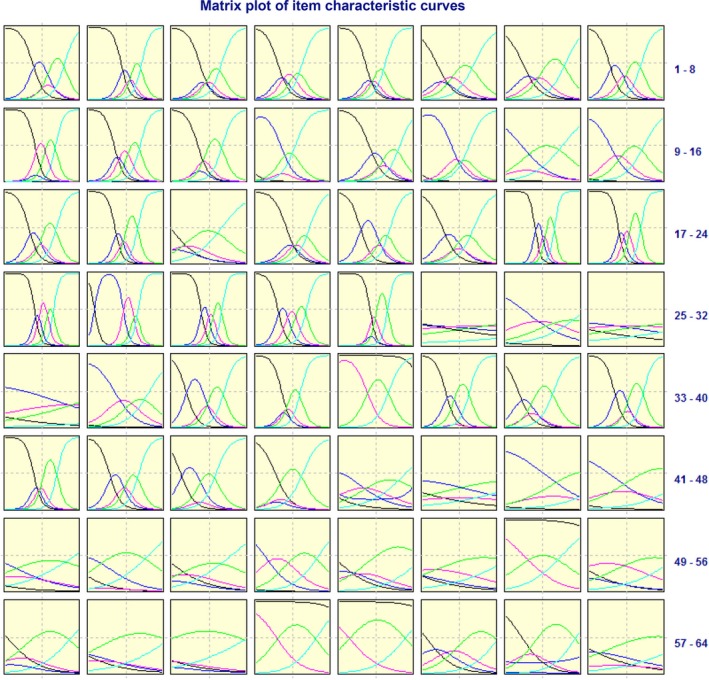
ICC matrix 1: Black, 2: Blue, 3: Magenta, 4: Green, 5: Cyan

The two‐step item selection process was based on CTT and IRT. This iterative process resulted in a final version comprised four domains, 11 subdomains and 54 items. For the first phase, statistical results for the items are given in Table 3. As we can see, 10 items (PHD15, PHD16, PSD14, PSD15, PSD16, PSD20, SOD4, SOD8, SOD9, THA8) were deleted based on the criteria described in the Methods. To ensure the reliability and validity of each item, we used the same methods to re‐select items and considered their practical significance based on the experts’ opinions for each item. All items remained after this process. Figure 2 shows the matrix plot of the ICCs for each item. Ideally, the first ICC curve should decrease monotonously, the last curve should increase monotonously and the other curves follow a normal distribution. The closer the ICC distribution is to the ideal state, the more information it contains, and vice versa. As seen in Figure 2, most items were satisfactory. Therefore, the preliminary measure comprised four domains, 11 subdomains and 54 items (Appendix 2).

**Table 3:** Item selection outcome based on CTT and IRT

Subdomain	Item	IRT					SD	*t*	Factor Loading	CAID	CC	Retest Reliability	Outcome
		a	b1	b2	b3	b4							
SOM	PHD1	2.17	−0.78	0.27	0.64	1.84	1.172	0.001	0.756	0.902	0.634	0.769	√
	PHD2	2.97	−0.38	0.20	0.57	1.30	1.285	0.001	0.858	0.895	0.777	0.843	√
	PHD3	2.08	−0.78	−0.33	0.14	1.02	1.206	0.001	0.793	0.897	0.748	0.810	√
	PHD4	2.07	−1.21	−0.63	0.08	0.80	1.110	0.001	0.775	0.898	0.717	0.664	√
	PHD5	2.37	−0.87	−0.42	0.01	0.75	1.151	0.001	0.780	0.897	0.737	0.737	√
	PHD6	1.39	−1.88	−1.16	−0.26	1.21	1.022	0.001	0.654	0.900	0.661	0.780	√
	PHD7	1.45	−1.66	−0.75	0.10	1.85	1.016	0.007	0.663	0.900	0.678	0.691	√
	PHD8	2.18	−1.39	−0.46	0.16	1.23	1.022	0.001	0.425	0.901	0.642	0.725	√
	PHD9	3.12	−0.61	−0.50	0.26	1.10	1.038	0.001	0.546	0.897	0.739	0.702	√
	PHD10	2.61	−0.95	−0.42	0.27	1.19	1.018	0.330	0.544	0.903	0.598	0.784	√
	PHD11	2.18	−0.93	−0.67	−0.15	1.08	1.019	0.004	0.610	0.904	0.586	0.772	√
	PHD12	1.75	−4.25	−0.96	−0.72	0.23	0.882	0.001	0.734	0.904	0.565	0.715	√
	PHD13	1.75	−0.57	0.36	0.86	1.96	1.260	0.039	0.603	0.903	0.640	0.656	√
	PHD14	1.70	−4.46	−0.66	0.07	0.77	1.074	0.001	0.651	0.900	0.679	0.766	√
	PHD15	0.83	−7.42	−1.73	−0.99	1.62	0.960	0.008	0.405	0.797	0.625	0.741	×
	PHD16	1.30	−5.54	−1.33	−0.18	1.46	0.909	0.014	0.417	0.794	0.620	0.784	×
PHY	PHD17	2.37	−1.11	−0.34	0.10	1.17	1.073	0.103	0.815	0.693	0.855	0.760	√
	PHD18	3.29	−0.89	−0.35	0.03	0.99	1.009	0.003	0.794	0.718	0.809	0.828	√
	PHD19	0.87	−3.21	−2.35	−1.22	1.04	1.004	0.023	0.811	0.741	0.765	0.775	√
WOR	PSD1	1.92	−0.52	0.03	0.57	1.40	1.354	0.001	0.678	0.833	0.881	0.822	√
	PSD2	2.17	−1.30	−0.04	0.42	1.17	1.171	0.030	0.790	0.802	0.876	0.828	√
	PSD3	1.59	−1.41	−0.35	0.16	1.19	1.211	0.001	0.792	0.770	0.896	0.751	√
ANX	PSD4	4.43	−0.65	−0.09	0.26	0.95	1.102	0.001	0.682	0.912	0.782	0.717	√
	PSD5	3.89	−0.68	−0.21	0.30	1.18	1.026	0.001	0.771	0.904	0.833	0.729	√
	PSD6	4.28	−0.64	−0.22	0.41	0.93	1.062	0.004	0.849	0.901	0.857	0.662	√
	PSD7	4.04	−2.56	−0.20	0.59	1.02	1.003	0.001	0.848	0.906	0.820	0.719	√
	PSD8	4.04	−0.67	−0.08	0.37	1.04	1.090	0.001	0.861	0.901	0.859	0.713	√
	PSD9	2.87	−1.20	−0.42	0.29	1.13	0.980	0.005	0.727	0.911	0.775	0.751	√
	PSD10	3.99	−0.50	−0.37	0.04	0.96	1.029	0.002	0.782	0.907	0.812	0.698	√
DEP	PSD11	0.16	−8.32	−1.11	5.87	16.96	1.194	0.001	0.717	0.645	0.781	0.709	√
	PSD12	0.55	−9.25	−1.57	0.97	3.62	1.015	0.001	0.765	0.661	0.734	0.750	√
	PSD13	0.20	−9.58	−2.45	3.21	10.47	1.207	0.001	0.786	0.587	0.831	0.756	√
	PSD14	0.28	−9.30	−0.05	4.52	13.33	0.938	0.001	0.428	0.743	0.602	0.700	×
	PSD15	1.14	−5.97	−0.91	0.48	1.93	0.987	0.046	0.594	0.775	0.655	0.736	×
	PSD16	2.24	−1.88	−0.45	0.09	1.22	0.984	0.001	0.440	0.773	0.662	0.740	×
FEA	PSD17	2.70	−0.90	−0.60	−0.21	0.65	1.018	0.004	0.684	0.743	0.757	0.635	√
	PSD18	1.77	3.90	−4.44	−0.75	1.05	1.027	0.087	0.726	0.765	0.695	0.711	√
	PSD19	2.45	−1.14	−0.38	−0.32	0.82	1.058	0.001	0.726	0.726	0.806	0.816	√
	PSD20	1.74	−2.02	−1.09	−0.64	0.86	0.890	0.422	0.550	0.780	0.618	0.851	×
SOC	SOD1	2.53	−0.98	−0.08	0.28	1.40	1.084	0.001	0.833	0.735	0.791	0.827	√
	SOD2	3.04	−0.71	−0.31	0.09	1.20	1.032	0.001	0.792	0.738	0.780	0.822	√
	SOD3	2.25	−1.33	−0.39	0.15	1.08	1.060	0.001	0.773	0.763	0.734	0.874	√
	SOD4	1.80	−2.33	−0.86	−0.64	0.56	0.940	0.264	0.405	0.788	0.648	0.874	×
	SOD5	1.57	−1.41	−1.15	−0.80	0.82	1.015	0.050	0.554	0.753	0.748	0.893	√
SUP	SOD6	0.68	−5.46	−1.96	−0.16	2.43	1.027	0.001	0.508	0.799	0.512	0.824	√
	SOD7	0.28	−7.28	−1.09	1.25	7.27	1.228	0.001	0.696	0.736	0.791	0.776	√
	SOD8	0.54	−9.14	0.12	1.51	5.90	0.992	0.022	0.701	0.783	0.587	0.728	×
	SOD9	0.54	−8.46	−1.31	0.57	5.31	0.939	0.330	0.779	0.801	0.479	0.766	×
	SOD10	0.44	−8.62	−3.33	−1.42	2.73	1.033	0.202	0.807	0.747	0.744	0.650	√
	SOD11	0.67	−5.10	−2.01	−1.75	1.85	1.033	0.122	0.896	0.732	0.803	0.850	√
	SOD12	0.56	−3.91	−2.84	−1.14	1.83	1.175	0.019	0.780	0.755	0.727	0.830	√
COM	THA1	0.98	−6.64	−2.25	−0.25	2.16	0.811	0.027	0.796	0.811	0.805	0.717	√
	THA2	0.66	−3.60	−1.88	−0.36	3.99	1.021	0.057	0.879	0.783	0.860	0.789	√
	THA3	0.39	−6.45	−3.81	−0.62	4.60	1.054	0.010	0.743	0.815	0.824	0.838	√
	THA4	0.71	6.34	−8.80	−1.54	1.56	1.026	0.116	0.722	0.808	0.829	0.700	√
AVA	THA5	0.48	−4.97	−3.29	0.18	4.46	1.021	0.067	0.797	0.687	0.813	0.751	√
	THA6	0.77	−2.88	−2.25	−1.10	2.41	1.035	0.001	0.753	0.705	0.794	0.924	√
	THA7	0.47	−5.48	−3.02	−1.52	3.81	1.052	0.134	0.790	0.680	0.823	0.776	√
	THA8	0.38	−7.09	−5.74	−3.76	3.28	0.944	0.378	0.569	0.795	0.652	0.819	×
SAT	THA9	1.01	5.35	−6.70	−1.30	1.95	1.005	0.030	0.732	0.801	0.745	0.860	√
	THA10	0.82	6.24	−7.88	−2.20	2.45	1.002	0.009	0.856	0.759	0.847	0.700	√
	THA11	0.89	−2.74	−1.21	0.24	3.23	1.004	0.163	0.81	0.773	0.815	0.729	√
	THA12	1.02	−1.72	−1.64	−0.56	2.72	1.040	0.001	0.775	0.793	0.768	0.802	√
	THA13	0.38	−4.82	−1.56	−0.39	5.63	1.249	0.001	0.657	0.841	0.706	0.826	√

“CC” is the abbreviation of Correlation Coefficient. × represents the item considered to be deleted, and √ considered to be kept.

### 3.4 Validation of the measure

The reliability, validity, DIF and feasibility of the 54 items were assessed, and the results are presented in the followed.

### 3.5 Reliability

Cronbach's α coefficient is an important indicator for the reliability. In general, its value should more than 0.70. For our PU‐PROM, the Cronbach's α of each subdomain was more than 0.800 for the measure and ranged from 0.817 to 0.907 for the four domains, indicating the measure was reliable (Table 4). In addition, we conducted a repeated survey of 50 patients, and the correlation coefficients of each item were >0.60, which showed the measure has high retest reliability.

**Table 4:** Cronbach's α coefficient of four domains and total measure

Domain	Subdomain	Cronbach's α coefficient
Physical	SOM	0.897
	PHY	0.854
Psychological	WOR	0.907
	ANX	0.884
	DEP	0.884
	FEA	0.905
Social	SOC	0.901
	SUP	0.817
Treatment	COM	0.844
	AVA	0.881
	SAT	0.871

### 3.6 Validity

The measure was also found to have good content validity as it allows direct communication with patients^26^, ^27^ and they thought the PU‐PROM was easy to understand and response. In the process of the item building and modifying phase, experts also agreed the measure was reasonable and comprehensive in content. We conducted CFA for the 54 items to investigate the factor structure of the measure. We also found that the indices of fit (GFI, RMR, NFI, NNFI, CFI, IFI) met the expected structure. On the whole, GFI, NFI, NNFI, CFI and IFI are all more than 0.90, and RMR is <0.09. The results are shown in Tables 5 and 6. The measure was able to distinguish between patients with peptic ulcers and controls. Our analysis of the different average scores using the independent two‐sample *t*‐test showed that all *P*‐values were <.05, indicating the measure had a good degree of differentiation (Table 7). As controls did not receive treatment and could not answer items in the treatment domain, no comparison was made for the SAT (satisfaction) subdomain.

**Table 5:** Goodness‐of‐fit statistics of CFA for PU‐PROM

	GFI	RMR	NFI	NNFI	CFI	IFI
PHD	0.81	0.100	0.88	0.88	0.90	0.90
PSD	0.93	0.056	0.95	0.96	0.97	0.97
SOD	0.98	0.038	0.98	0.98	0.99	0.99
THA	0.94	0.045	0.95	0.95	0.96	0.96
Total	0.80	0.072	0.86	0.91	0.92	0.92

**Table 6:** Maximum likelihood estimation of CFA for PU‐PROM

Domain	Subdomain	Item	Factor loading	SE	*t*	*R* ^2^	Error variance
PHD	SOM	PHD1	0.69	0.05	14.83	0.39	0.73
		PHD2	0.90	0.05	17.24	0.50	0.83
		PHD3	0.76	0.06	13.61	0.34	1.11
		PHD4	0.76	0.05	14.30	0.37	0.98
		PHD5	0.91	0.05	17.03	0.49	0.88
		PHD6	0.85	0.05	16.05	0.44	0.90
		PHD7	0.67	0.05	14.21	0.37	0.77
		PHD8	0.64	0.05	13.83	0.35	0.75
		PHD9	0.76	0.05	15.37	0.42	0.80
		PHD10	0.64	0.05	13.47	0.34	0.82
		PHD11	0.63	0.05	13.42	0.33	0.79
		PHD12	0.68	0.05	13.70	0.35	0.87
		PHD13	0.71	0.05	13.84	0.35	0.93
		PHD14	0.81	0.06	13.96	0.36	1.18
	PHY	PHD15	1.04	0.04	23.11	0.81	0.25
		PHD16	0.80	0.04	17.88	0.54	0.54
		PHD17	0.89	0.04	20.00	0.65	0.43
PSD	WOR	PSD1	1.03	0.05	22.38	0.71	0.44
		PSD2	1.16	0.05	25.16	0.83	0.28
		PSD3	1.23	0.05	24.04	0.78	0.43
	ANX	PSD4	1.02	0.05	19.96	0.61	0.67
		PSD5	1.08	0.05	21.70	0.68	0.55
		PSD6	0.96	0.04	22.98	0.73	0.34
		PSD7	0.95	0.05	20.76	0.64	0.50
		PSD8	0.84	0.05	17.19	0.49	0.73
		PSD9	0.68	0.05	13.20	0.32	0.96
		PSD10	0.60	0.05	12.12	0.28	0.94
	DEP	PSD11	0.95	0.05	20.94	0.66	0.46
		PSD12	0.89	0.04	21.72	0.70	0.34
		PSD13	1.09	0.05	23.76	0.79	0.31
	FEA	PSD14	1.00	0.04	24.81	0.81	0.23
		PSD15	0.96	0.04	25.10	0.82	0.20
		PSD16	1.07	0.05	22.24	0.70	0.49
SOD	SOC	SOD1	0.94	0.04	20.92	0.65	0.47
		SOD2	0.99	0.04	23.75	0.77	0.30
		SOD3	0.96	0.04	23.13	0.74	0.32
		SOD4	0.99	0.05	20.84	0.65	0.54
	SUP	SOD5	0.65	0.05	13.44	0.35	0.79
		SOD6	0.95	0.05	18.70	0.59	0.64
		SOD7	0.83	0.04	19.94	0.64	0.39
		SOD8	0.55	0.05	10.57	0.23	0.98
		SOD9	0.93	0.05	20.05	0.65	0.47
THA	COM	THA1	0.67	0.04	16.14	0.46	0.53
		THA2	0.65	0.05	13.73	0.36	0.75
		THA3	0.98	0.04	22.33	0.75	0.32
		THA4	0.97	0.04	21.80	0.72	0.36
	AVA	THA5	0.82	0.04	20.33	0.64	0.38
		THA6	0.93	0.04	23.73	0.80	0.22
		THA7	0.89	0.04	21.71	0.70	0.33
	SAT	THA8	0.63	0.05	13.96	0.36	0.71
		THA9	0.89	0.04	23.34	0.76	0.25
		THA10	0.87	0.04	22.71	0.73	0.28
		THA11	0.74	0.04	18.37	0.55	0.46
		THA12	0.75	0.04	18.32	0.54	0.48

**Table 7:** Scores comparison between peptic ulcer patients and controls

Subdomain	Peptic Ulcer	Controls	*t*/*t′*	*P*
SOM	47.07±11.02	61.50±2.08	−27.197	<.001
PHY	11.93±2.96	13.31±0.92	−8.781	<.001
WOR	9.77±3.59	12.12±1.22	−12.046	<.001
ANX	23.88±6.49	30.26±1.47	−19.869	<.001
DEP	9.96±3.12	12.66±1.07	−15.866	<.001
FEA	11.57±3.17	13.36±0.96	−10.763	<.001
SOC	14.26±4.08	16.93±1.31	−12.219	<.001
SUP	19.03±4.33	21.39±1.42	−10.074	<.001

### 3.7 DIF

This study used a mean and covariance structure (MACS) model based on sex for the DIF analysis, through which we examined whether there were differences between men and women. We performed DIF analysis for the 11 subdomains by sex (Table 8). As the subdomains including: physical status (PHY), work stress (WOR), depression (DEP), fear (FEA) and availability (AVA) all contained only three items, their degree of freedom was 0, which cannot be well fitted the “baseline model,” so in the study, items contained in these subdomains did not exist DIF. For the subdomain of “social adaptation (SUP),” the result suggested that there are differences in model fitting, items exist DIF, but modification index (MI) prompted the item difficulty parameters and discrimination parameters are cross gender equal. The results showed there is not significant DIF. Other subdomains of DIF analysis results are shown that the items of the PU‐PROM do not exist DIF.

**Table 8:** Transgender DIF nested model results

		χ^2^	*df*	GFI	RMR	RMSEA	NFI	NNFI	CFI	IFI	Δχ^2^	Δ*df*
SOM	Measurement equivalence model	1032.561	195	0.806	0.123	0.132	0.869	0.901	0.894	0.894		
	Baseline model	1001.553	154	0.805	0.108	0.150	0.873	0.872	0.892	0.892	31.008	41
		MI1=2.662, *P*>.05/14					MI2=2.211, *P*>.05/13				*P*>.05	
PHY	Measurement equivalence model	14.423	8	0.972	0.143	0.0572	0.976	0.991	0.989	0.988		
WOR	Measurement equivalence model	10.238	8	0.985	0.0595	0.0338	0.988	0.998	0.997	0.997		
ANX	Measurement equivalence model	170.847	48	0.910	0.105	0.102	0.941	0.962	0.957	0.956		
	Baseline model	154.355	10	0.924	0.0703	0.136	0.946	0.933	0.955	0.956	16.492	38
		MI1=4.288, *P*>.05/7					MI2=1.082, *P*>.05/6				*P*>.05	
DEP	Measurement equivalence model	12.247	8	0.985	0.106	0.0465	0.984	0.995	0.994	0.994		
FEA	Measurement equivalence model	9.313	8	0.980	0.0678	0.0259	0.989	0.999	0.998	0.998		
SOC	Measurement equivalence model	21.438	15	0.970	0.0525	0.0419	0.985	0.997	0.996	0.996		
	Baseline model	17.676	4	0.973	0.0303	0.118	0.988	0.973	0.991	0.991	3.762	11
		MI1=0.743, *P*>.05/4					MI2=1.321, *P*>.05/3				*P*>.05	
SUP	Measurement equivalence model	53.386	24	0.958	0.0869	0.0707	0.954	0.978	0.974	0.974		
	Baseline model	28.690	10	0.975	0.0501	0.0873	0.975	0.966	0.983	0.983	24.696	14
		MI1=2.099, *P*>.05/5					MI2=2.216 (SOD6), *P*>.05/4				*P* <.05	
	Partial measurement equivalence model	53.412	23	0.958	0.0870	0.0735	0.954	0.977	0.973	0.973	−0.026	1
		MI3=2.134, *P*>.05/4					MI4=2.215, *P*>.05/4				*P*>.05	
COM	Measurement equivalence model	100.327	15	0.892	0.0957	0.152	0.897	0.928	0.910	0.910		
	Baseline model	91.545	4	0.899	0.0741	0.299	0.909	0.736	0.912	0.913	8.782	11
		MI1=0.780, *P*>.05/4					MI2=0.997, *P*>.05/3				*P*>.05	
AVA	Measurement equivalence model	8.934	8	0.983	0.0628	0.0218	0.987	0.999	0.998	0.998		
SAT	Measurement equivalence model	50.964	24	0.954	0.154	0.0677	0.968	0.985	0.982	0.982		
	Baseline model	36.804	10	0.971	0.0371	0.105	0.977	0.965	0.983	0.983	14.16	14
		MI1=0.417, *P*>.05/5					MI2=1.411, *P*>.05/4				*P*>.05	

Modification index 1 (MI1) represents the largest fixed index associated with the intercept, and MI2 is associated with the load of the factors. MI3, MI4 represent the above interpretation after the item “SOD6” being removed, respectively.

### 3.8 Feasibility

The acceptance rate and completion rate of the measure were beyond 85%, and the average completion time was within 15 minutes, indicating that the measure was feasible.

Therefore, the final PU‐PROM is comprised of four domains, 11 subdomains and 54 items (Appendix 3) ,and the theoretical framework is shown in the Table 9.

**Table 9:** Construction frame of final PU‐PROM

Domain	Subdomain	Item
Physical domain (PHD)	Physical symptoms (SOM)	1−, 2−, 3−, 4−, 5−, 6−, 7−, 8−, 9−, 10−, 11−, 12−, 13, 14−
	Physical status (PHY)	15−, 16−, 17−
Psychological domain (PSD)	Work stress (WOR)	1−, 2−, 3−
	Anxiety (ANX)	4−, 5−, 6−, 7−, 8−, 9−, 10−
	Depression (DEP)	11−, 12, 13
	Fear (FEA)	14−, 15−, 16−
Social domain (SOD)	Social support (SOC)	1−, 2−, 3−, 4−
	Social adaptation (SUP)	5, 6, 7, 8, 9
Treatment domain (THA)	Compliance (COM)	1, 2, 3, 4
	Availability (AVA)	5, 6, 7
	Satisfaction (SAT)	8−, 9, 10, 11, 12

“−”means negative item, others means positive item.

## 4 DISCUSSION

Peptic ulcers have various causes and mainly occur in the stomach and duodenum mucosa. A peptic ulcer is a chronic disease characterized by high incidence, low mortality, spontaneous remission and periodical paroxysms. The main peptic ulcer complications are haemorrhage and perforation. The disease leads to a decrease in productivity and brings significant loss to society, and the treatment cost contributes to social and economic burdens. Therefore, a PRO measure for peptic ulcers may improve patients’ QoL and can be used to evaluate clinical curative effect.

Patients are increasingly involved in evaluations of health‐care quality.^28^, ^29^, ^30^ Internationally, many HRQoL measures have been developed and applied in chronic diseases of the digestive system. However, existing measures have some deficiencies. For example, the NHP focuses on a more serious disability level and is not sensitive to relatively mild condition changes; the SIP has wide coverage, but is very long; and the SF‐36 is widely used, but does not identify specific areas of the disease. The QPD is restricted to functional dyspepsia patients. The UESS involves fewer items and uses a 100 mm visual record to show the degree of symptom severity; higher scores indicate more serious symptoms. The PUDQ only involves three aspects of ulcers (pain, mood and social function) and does not accurately reflect the status of the disease. The QLDUP only reflects QOL in duodenal ulcer patients. In addition, at present, these questionnaires are mainly focused on physiology, psychology and society, and are limited.

Our peptic ulcer PRO measure (PU‐PROM) has improved on the disadvantages of the above tools. This is a specific measure for peptic ulcers. It has a high correlation with the disease, and the results can reflect the actual life‐experience of patients. Our PU‐PROM also includes a new and significant concept of a treatment domain, which is comprehensive, and does not only consider the HRQoL index in the traditional sense. By measuring the patient's satisfaction, compliance and side‐effects of drugs, our measure also provides a basis for clinical treatment, new drug research and development, and clinical review.

In developing the measure, we used cognitive investigation to form the conceptual framework, CTT and IRT to select items, and CFA to validate items. The United States FDA has emphasized the importance of clinical outcomes and provided guidance for the establishment of PRO measures.^31^ This study was based on FDA guidance, and our methods of investigation strictly followed the established PRO measure production process. We used expert and patient opinions and suggestions to build an initial measure. A sample investigation that combined CTT and IRT was used to select items. After a large‐scale formal investigation, we evaluated the reliability, validity, DIF and feasibility of the measure, finalizing a PRO measure for patients with peptic ulcers that included four domains, 11 subdomains and 54 items.

A standard operating procedure was used to: generate appropriate items and domains; ensure the instrument was comprehensive relative to its intended measurement concept, population and context of use; and ensure patient understanding of the instrument (i.e, instructions, items and response options).^32^

In contrast to previous assessments of measures, we directed attention to the items rather than the structure validity of the measure. Items for the PU‐PROM were based on a review of the literature and other related questionnaires, face‐to‐face interviews with patients and discussions with expert professionals. The content validity index further strengthened the content validity of our preliminary measure, as in the development process, we selected items by this index. The role of content validity index in the process of preparation and evaluation cannot be ignored, and can meet the quality requirements of the measure before implementation.

In the development stage, we used CTT and IRT to select the items, striving for items that had strong representativeness, independence and high sensitivity. CTT is easier to understand and is more commonly used than IRT. However, it neglects the estimation of the respondent's ability, and the reliability of the results is inadequate. Compared with the CTT, IRT has some advantages including: the estimate of the respondent's ability is independent of the item; the difficulty parameter of the items and respondent's ability is unified; parameter estimation of items is independent; and the accuracy of the estimator of the respondent's ability can be estimated. Use of the IRT enriched the representativeness, independence, internal consistency, sensitivity, fairness and importance of the items. However, the results of the measure should also be considered in combination with clinical knowledge and the patient's illness and psychological state, to ensure the quality of the measure.

Differential item functioning is a research hotspot in the field of psychology and education measurement. DIF is more commonly used in the detection of fairness and effectiveness of examination questions. This study introduced DIF into the process of developing a clinical measure and broadened the application scope of DIF. The influence of DIF on the content of the measure provided a new method for item selection and content evaluation, and improved the quality of the developed measure.

The PU‐PROM we developed fills a gap in clinical outcome measurement for patients with peptic ulcers, and addresses the deficiencies in existing tools. It will also provide valuable data for new drug development for peptic ulcers, because PROs are increasingly included as key primary or secondary end points in clinical studies to support drug development.^33^

However, there were some limitations in this study that should be resolved and improved in the future research. First, there were two clinical investigations during the development of the measure, and the results might have been affected by the investigation environment to produce bias. Respondents and investigators might have also affected the results of the study. Second, we did not use criterion validity to measure the PRO measure. As the content of the measure is various, and considering the patients’ physical and psychological states, we did not choose a well‐established measure as a criterion to test consistency. Third, the PRO measure scores were calculated using a 5‐point Likert scale, but there is no clear and suitable method to evaluate the overall QoL. Forth, the sample populations may not fully represent the entire population of patients with peptic ulcer. Our participants were from only the Shanxi province in northern China. Therefore, future research should explore the reliability and validity of the PU‐PROM with a nationwide sample. The PU‐PROM was administrated to native‐Chinese speaking individuals. Therefore, further work is needed to conduct cross‐cultural adaptation in different countries. Fifth, we performed DIF analysis for the 11 subdomains by sex, which showed sex had no influence on peptic ulcer, but we did not analyse age. We should consider the effect of age in the next application and development of the PU‐PROM. In addition, there are no standard quantitative indicators to support clinical decision making for the corresponding disease. Finally, we did not consider the demands of DIF analysis in the design phase, which made the numbers of items in some subdomains too small, meaning that in the process of DIF analysis, a “baseline model” could not be implemented. Additional studies should further expand the scope as well as the number of participants. We should also compare our PU‐PROM with a criterion measure and assess the measure in terms of the range, reliability, validity and applicability.

## 5 CONCLUSIONS

Our study makes important contributions to the treatment and outcomes of patients with peptic ulcers and develops a specific measurement instrument for this group. We found strong evidence for the reliability and validity of our PU‐PROM. However, we do not consider that our PU‐PROM is able to replace other related questionnaires. Therefore, we need to further improve the measure to ensure it suitable for a wider range of people. We could also extend the measure for other uses such as patients’ health conditions, clinical effects evaluation, new drug development, health service deployment and clinical research.

## CONFLICT OF INTEREST

The authors declare that they have no conflict of interest.

## HUMANS AND ANIMAL RIGHTS

All procedures performed in studies involving human participants were in accordance with the ethical standards of the institutional and/or national research committee and with the 1964 Helsinki Declaration and its later amendments or comparable ethical standards.

## INFORMED CONSENT

Informed consent was obtained from all individual participants included in the study.

## APPENDIX 1 Formation of PU‐PROM item pool of 64

### 1.1

**Table:**

Item	Item
PHD1. Do you have epigastric pain?	PSD14. Are you optimistic about the future?
PHD2. Does the pain change with food?	PSD15. Are you worried about bringing harm to yourself or a loved one?
PHD3. Is the pain getting worse when you are hungry?	PSD16. Are you feeling fine about everything? Nothing bad will happen, right?
PHD4. Is the pain getting worse after meals?	PSD17. Do you feel frustrated and hopeless about your illness?
PHD5. Do you have epigastric pain during the night?	PSD18. Do you feel the illness is a burden of your family?
PHD6. Is the epigastric pain regular?	PSD19. I don't want to talk about my illness to other people.
PHD7. Do you have abdominal distension?	PSD20. I am not interested in social activities any more.
PHD8. Do you have heartburn in your stomach?	SOD1. Is your job affected by your illness?
PHD9. Do you have acid regurgitation?	SOD2. Are your hobbies affected by your illness (such as playing chess, dancing)?
PHD10. Do you have eructation?	SOD3. Does your illness prevent you from taking family responsibilities?
PHD11. Do you feel nauseated?	SOD4. Does your illness estrange you from your relatives, neighbours and friends?
PHD12. Do you have hematemesis?	SOD5. Does your illness prevent you from participating the social and family activities?
PHD13. Is your bowel movement OK?	SOD6. My family is concerned about my illness.
PHD14. Have you noticed any changes in the colour of your faeces, for example, are they getting darker?	SOD7. My relatives, neighbours and friends all care about my illness.
PHD15. Do you feel dizzy?	SOD8. My relatives, neighbours and friends all give me material help and support.
PHD16. Do you feel general weakness?	SOD9. My relatives, neighbours and friends all give me spiritual help and support.
PHD17. Do you feel fatigue?	SOD10. My family gives me material help and support.
PHD18. Do you have loss of appetite?	SOD11. My family gives me spiritual help and support.
PHD19. Have you lost any weight?	SOD12. The family often reminds me of medication.
PSD1. Do you do repetitive work?	THA1. Take my medicine as instructed.
PSD2. Is your work trivial, complicated and big workload?	THA2. I follow my doctor's instruction and get rid of my bad habits.
PSD3. Does your job have requirement on hours and speed?	THA3. I follow my doctor's instruction and do regular follow‐ups.
PSD4. Are you stressed or nervous than usual?	THA4. All of the checks and tests about my illness are necessary.
PSD5. Are you more irritable?	THA5. Treatment is effective for my illness..
PSD6. Do you get angry more easily than usual?	THA6. My mood improved after treatment.
PSD7. Do you feel unhappy and depressed?	THA7. I have physical improvement after treatment.
PSD8. Are you worried about the epigastric pain?	THA8. My life confidence increased after treatment.
PSD9. Is your sleep affected by the pain?	THA9. Are you worried about the side‐effects of your medicine?
PSD10. Are you bothered by your indigestion?	THA10. The doctors are friendly to me.
PSD11. Do you feel it difficult to concentrate to do one thing?	THA11. Are you satisfied with the medical services?
PSD12. Do you have no difficulty in doing your daily activities?	THA12. Are you satisfied with the costs of medical care?
PSD13. Are you still interested in the activities you love to do?	THA13. Are you satisfied with current treatment?

## APPENDIX 2 Primary PU‐PROM item pool of 54

### 2.1

**Table:**

Item	Item
PHD1. Do you have epigastric pain?	PSD11. Do you feel it difficult to concentrate to do one thing?
PHD2. Does the pain change with food?	PSD12. Do you have difficulty in doing your daily activities?
PHD3. Is the pain getting worse when you are hungry?	PSD13. Are you still interested in the activities you love to do?
PHD4. Is the pain getting worse after meals?	PSD14. Do you feel frustrated and hopeless about your illness?
PHD5. Do you have epigastric pain during the night?	PSD15. Do you feel the illness is a burden of your family?
PHD6. Is the epigastric pain regular?	PSD16. I don't want to talk about my illness to other people.
PHD7. Do you have abdominal distension?	SOD1. Is your job affected by your illness?
PHD8. Do you have heartburn in your stomach?	SOD2. Are your hobbies affected by your illness (such as playing chess, dancing)?
PHD9. Do you have acid regurgitation?	SOD3. Does your illness prevent you from taking family responsibilities?
PHD10. Do you have eructation?	SOD4. Does your illness estrange you from participating the social and family activities?
PHD11. Do you feel nauseated?	SOD5. My family is concerned about my illness.
PHD12. Do you have hematemesis?	SOD6. My relatives, neighbours and friends all care about my illness.
PHD13. Is your bowel movement OK?	SOD7. My family gives me material help and support.
PHD14. Have you noticed any changes in the colour of your faeces, for example, are they getting darker?	SOD8. My family gives me spiritual help and support.
PHD15. Do you feel fatigue?	SOD9. The family often reminds me of medication.
PHD16. Do you have loss of appetite?	THA1. Take my medicine as instructed.
PHD17. Have you lost any weight?	THA2. I follow my doctor's instruction and get rid of my bad habits.
PSD1. Do you do repetitive work?	THA3. I follow my doctor's instruction and do regular follow‐ups.
PSD2. Is your work trivial, complicated and big workload?	THA4. All of the checks and tests about my illness are necessary.
PSD3. Does your job have requirement on hours and speed?	THA5. Treatment is effective for my illness.
PSD4. Are you stressed or nervous than usual?	THA6. My mood improved after treatment.
PSD5. Are you more irritable?	THA7. I have physical improvement after treatment.
PSD6. Do you get angry more easily than usual?	THA8. Are you worried about the side‐effects of your medicine?
PSD7. Do you feel unhappy and depressed?	THA9. The doctors are friendly to me.
PSD8. Are you worried about the epigastric pain?	THA10. Are you satisfied with the medical services?
PSD9. Is your sleep affected by the pain?	THA11. Are you satisfied with the costs of medical care?
PSD10. Are you bothered by your indigestion?	THA12. Are you satisfied with current treatment?

## APPENDIX 3 Final version of PU‐PROM

### 3.1

**Table:**

	Never	Occasionally	About half of the time	Often	Almost everyday
A. Physical domain					
1. I had epigastric pain	0	1	2	3	4
2. I felt the pain change with food	0	1	2	3	4
3. I thought the pain getting worse when I am hungry	0	1	2	3	4
4. I thought the pain getting worse after meals	0	1	2	3	4
5. I had epigastric pain during the night	0	1	2	3	4
6. I had epigastric pain regular	0	1	2	3	4
7. I had abdominal distension	0	1	2	3	4
8. I had heartburn in my stomach	0	1	2	3	4
9. I had acid regurgitation	0	1	2	3	4
10. I had eructation	0	1	2	3	4
11. I felt nausea	0	1	2	3	4
12. I had hematemesis	0	1	2	3	4
13. I thought my bowel movement is OK	0	1	2	3	4
14. My face looked dark and dull	0	1	2	3	4
15. I felt fatigue	0	1	2	3	4
16. I felt loss of appetite	0	1	2	3	4
17. I was losing weight	0	1	2	3	4

	Never	Occasionally	About half of the time	Often	Almost everyday
B. Psychological domain					
1. Doing repetitive work	0	1	2	3	4
2. The work is trivial, complicated and big workload	0	1	2	3	4
3. Work has requirement on hours and speed	0	1	2	3	4
4. I felt stressed or nervous than usual	0	1	2	3	4
5. I felt more irritable	0	1	2	3	4
6. I got angry more easily than usual	0	1	2	3	4
7. I felt unhappy and depressed	0	1	2	3	4
8. I was worried about the epigastric pain	0	1	2	3	4
9. My sleep was affected by the pain	0	1	2	3	4
10. I was bothered by indigestion	0	1	2	3	4
11. I felt it difficult to concentrate to do one thing	0	1	2	3	4
12. I have difficulty in doing my daily activities	0	1	2	3	4
13. I felt interested in the activities I love to do	0	1	2	3	4
14. I felt frustrated and hopeless about my illness	0	1	2	3	4
15. I thought the illness is a burden of my family	0	1	2	3	4
16. I didn't want to talk about my illness to other people	0	1	2	3	4

	Never	Occasionally	About half of the time	Often	Almost everyday
C. Social domain					
1. My job affected by the illness	0	1	2	3	4
2. My hobbies affected by the illness	0	1	2	3	4
3. My illness prevent me from taking family responsibilities	0	1	2	3	4
4. My illness estrange me from participating the social and family activities	0	1	2	3	4
5. My family is concerned about my illness	0	1	2	3	4
6. My relatives, neighbours and friends all care about my illness	0	1	2	3	4
7. My family gives me material help and support	0	1	2	3	4
8. My family gives me spiritual help and support	0	1	2	3	4
9. The family often reminds me of medication	0	1	2	3	4

	Never	Occasionally	About half of the time	Often	Almost everyday
D. Treatment domain					
1. Take medicine as instructed	0	1	2	3	4
2. Follow doctor's instruction and get rid of bad habits	0	1	2	3	4
3. Follow doctor's instruction and do regular follow‐ups	0	1	2	3	4
4. I thought the checks and tests are necessary	0	1	2	3	4
5. I thought my treatment is effective for my illness	0	1	2	3	4
6. My mood improved after treatment	0	1	2	3	4
7. I have physical improvement after treatment	0	1	2	3	4
8. I was worried about the side‐effects of my medicine	0	1	2	3	4
	**Very dissatisfaction**	**Dissatisfaction**	**General satisfaction**	**Satisfaction**	**Very satisfaction**
9. The doctors are friendly to me	0	1	2	3	4
10. I was satisfied with the medical services	0	1	2	3	4
11. I was satisfied with the costs of medical care	0	1	2	3	4
12. I was satisfied with current treatment	0	1	2	3	4
